# Effects of *Periplaneta americana* extracts on the growth and proliferation of cutaneous interstitial cells in cutaneous-wound healing

**DOI:** 10.3389/fphar.2022.920855

**Published:** 2022-08-29

**Authors:** Zheng Liang, Guiqi Han, Zecheng Luo, Baojie Li, Wentao Liu, Chongyang Shen

**Affiliations:** ^1^ Chengdu University of Traditional Chinese Medicine, Chengdu, China; ^2^ The First Affiliated Hospital of Chengdu University of Traditional Chinese Medicine, Chengdu, China

**Keywords:** cutaneous wound healing, *Periplaneta americana* extracts, Stat3 signaling pathway, KFX, mesenchymal stromal cells

## Abstract

Cutaneous-wound healing requires a coordinated reaction of multiple cells, including interstitial cells. Impaired recovery of cutaneous wounds can lead to various adverse health outcomes. Kangfuxin (KFX), an extract obtained from *Periplaneta americana,* is beneficial in cutaneous-wound healing. In this study, we isolated dermal cells from suckling mice and established a mouse model of cutaneous injury to evaluate the therapeutic effects of KFX. Cell biology experiments indicated that treatment with KFX improved cell proliferation and migration and also repaired cutaneous wounds in the animal model. Activation of the signal transducer and activator of transcription 3 (STAT3) signaling pathway was the core molecular mechanism of KFX. Our study provides a theoretical and practical basis for the clinical application of KFX in cutaneous-wound healing.

## Introduction

The skin, the largest organ of the human body, includes the epidermis, dermis, subcutaneous-fat layer, and accessory organs. The epidermis is an epithelial-cell (EC) layer that functions as an essential barrier to protect the body from infection, tissue damage, and bodily-fluid loss (Blanpain and Fuchs, 2006). When the skin, the body’s first protective barrier, is breached, a series of physiological activities and metabolic reactions are activated to heal the cutaneous injury and re-establish the skin’s structural and functional integrity (Gonzales and Fuchs, 2017). However, defects in the cellular and molecular reactions of cutaneous-wound healing cause such healing to fail, resulting in acute and chronic cutaneous-trauma disorders (Wilson et al., 2017). Acute trauma, burns, and skin ulcers are severe clinical problems that burden society and patients and that consume vast medical resources (Martínez et al., 2016). To make matters worse, cutaneous wounds, one of the most common types of soft-tissue injuries, usually take a long time to heal. This, combined with environmental and human factors, frequently leads to severe deep cutaneous injuries or epidermal-tissue infections in affected patients (Opneja et al., 2019).

Cutaneous-wound healing is a spontaneous, highly organized, and strictly regulated physiological bodily activity that can effectively restore the integrity of the skin (Patteson et al., 2020). Wound healing is a multi-cellular collaborative process. First, a high rate of cell proliferation and rapid migration of fibroblasts and keratinocytes are needed to reconstruct skin in the cellular-system and extracellular-matrix (ECM) microenvironment. Second, cutaneous-wound healing requires proliferation of a great many vascular endothelial cells (VECs). The process embeds VECs into the wound to form new blood vessels, providing adequate nutrition for cellular-system reconstruction in the skin. (Zhao et al., 2017). Cutaneous-wound healing is typically divided into three overlapping stages: the inflammatory-reaction stage, the cell proliferation and reconstruction stage, and the cutaneous-maturation stage. Cell proliferation and reconstruction together form the most critical stage of cutaneous-wound healing and are responsible for the healing of these wounds or of skin tissue lesions (Wang et al., 2010).

Furthermore, cutaneous cells play a leading role in cutaneous-wound healing; their most vital contribution is stimulating the proliferative response of stem cells (Bianco, 2014). Since stem cell activation is the basis of cutaneous-wound healing, scientists have conducted many studies on the roles played by epidermal stem cells (ESCs) and hair follicle stem cells (HFSCs) in this process. These types of stem cells were found to be rapidly activated after cutaneous injury and to help re-epithelialize the wound surface and reconstruct skin accessory organs (Dekoninck and Blanpain, 2019). Different types of mesenchymal stem cells (MSCs) are distributed throughout different structural layers of the skin; they maintain the homeostasis of skin structure and function through continuous renewal and differentiation (An et al., 2015).

Traditional treatments for cutaneous wounds include skin grafting and artificial-replacement covering (Diao et al., 2019). However, due to the limited matching-donor skin resources available in clinical practice, immune rejection can occur in skin graft surgery, skin tissue infections can arise, and high-cost risks can occur in the treatment process. It is difficult for traditional treatments to meet these needs (Hu et al., 2019). Many treatments based on tissue growth factors (GFs) have excellent reparative effects on cutaneous wounds. However, biosafety must be considered due to the increased risks of unsatisfactory biocompatibility and cancer (Hwang et al., 2018).

*Periplaneta americana* (*P. americana*) is the largest insect in the cockroach family Blattellidae and one of the oldest and most active insect groups found worldwide (Song et al., 2017). Scientists have invested years of research and exploration into the bioactive substances and unique physiological mechanisms of *P. americana*, which has been used in traditional Chinese medicine (TCM) to eliminate blood stasis, remove blood accumulation, detoxify, promote urination, and reduce swelling. This species also provides alternative natural treatments for ulcers, burn wounds, tuberculosis, ulcerative colitis, heart disease, and cancer. In particular, it is widely used in TCM to treat various ulcers (Shi et al., 2021). The scientists referred to above have screened active compounds from *P. americana* for effectiveness in cutaneous-wound healing. Song et al. demonstrated that *P. americana* extracts can improve burn healing by activating the Janus-associated kinase (JAK)/signal transducer and activator of transcription 3 (STAT3) signaling pathway in keratinocytes (Li et al., 2019). Zhong et al. reported that these extracts can upregulate protein and messenger ribonucleic acid (mRNA) expression in irradiation injuries to skin (Zhang et al., 2013). Finally, Fu et al. showed that *P. americana* extract can help enhance cutaneous-fibroblast migration. These studies offer some evidence of the healing potential of this species.

Kangfuxin (KFX) is refined from the alcohol of *P. americana* extract (Peng et al., 2014). It has been approved by the China Food and Drug Administration for clinical wound treatment and is widely applied in the treatment of gastric ulcers, multiple colitis, pressure ulcers, and diabetic foot ulcers. Herein, we administered KFX to assess its healing effect in cutaneous-wound models *in vitro* and *in vivo* and to explore its potential molecular mechanisms. Based on previous research, in this study we attempted for the first time to develop an efficient, economical treatment for cutaneous-wound healing using KFX.

## Materials and methods

### Kangfuxin preparation

We purchased KFX from Xinglin Pharmacy (Chengdu, China).

### Isolation of dermal cells from suckling mice

After disinfecting the skin with 75% alcohol, we dislocated the necks of suckling female BALB/C mice (3 days old). Mouse thenar-skin samples were excised, tissues were rinsed with phosphate-buffered saline (PBS), and subcutaneous fat and blood vessels were carefully removed. Then, we incubated the tissues in 0.25% trypsin–EDTA and 2 mg/ml dispase (volume ratio, 1:1; MilliporeSigma, St. Louis, MO, United States) at 37°C for 1 h. After dispase treatment, we separated the epidermis, cut the dermal tissue into pieces of approximately 1.0 mm^3^ using surgical scissors, and digested these pieces with 0.25% collagenase II at 37°C for 2 h. The cell complex was centrifuged at 500*g* for 5 min. Finally, we collected the dermal cells and washed them three times in PBS to acquire dermal MSCs.

### Cell culture

MSCs from suckling mice were cultured in MedGro Stem Cell Medium supplemented with 10% fetal bovine serum (FBS; GIBCO) in a 37°C, 5% CO_2_ incubator.

### Animals

We purchased wild-type (WT) female BALB/c mice (6–8 weeks old, 23–27 g) from Cheng Du Dossy Animal Technology Co., Ltd. (Chengdu, China). They were maintained at the Experimental Animal Center of Basic Medical College of Chengdu University of TCM. Animals were treated in accordance with the regulations of the Experimental Animal Ethics Committee of Chengdu University of TCM (TCM-LAEC2020074). The mice drank water and ate freely, and the room was kept at a temperature of 22°C ± 2°C and humidity of 40% ± 10%.

### Cell counting kit-8 assay

Cells treated with different concentrations of KFX in 96-well plates at a density of 1 × 10^4^ cells per well. After 2 h of incubation, 10 μL CCK-8 solution were added to each well. Cells were further incubated for 1 h in a 37°C incubator. We obtained data using a microplate reader (Thermo Fisher Scientific, Waltham, MA, United States) with absorbance at 450 nm. ImagePro Plus statistical-analysis and plotting software version 6.0 (Media Cybernetics, Rockville, MD, United States) was used to analyze the experimental data and calculate cell viability and proliferation rates. After calculating the difference between the drug administration group and the blank control group, we compared the proliferation percentages of the two groups. The experiment was repeated three times, with five replicates per sample.

### Scratch wound assay

We performed a scratch wound assay by seeding target cells on 6-well plates, growing them to a cell density of 90%, and then starving the cells for 48 h in serum-free medium with or without different concentrations of KFX. The medium was removed, and the cells were scratched with a 200-µl pipette to form a uniform cell-free wound area. Cell debris was gently cleaned away with PBS buffer. At 0, 24, and 48 h, we observed and photographed cell migration to the wound area under a bio-electron microscope (ECLIPSE Ti-E; Nikon, Tokyo, Japan). Picture magnification was ×100. We used ImageJ software (National Institutes of Health [NIH], Bethesda, MD, United States) to analyze scratch widths at 0, 24, and 48 h of culture, and percentages of scratch width reduction were compared to assess cell mobility. The experiment was repeated three times with three replicates per sample.

### Cutaneous-perforation wound healing model

All mice were anesthetized by inhalation of avertin. We established a classic cutaneous excision wound model on the back of each mouse after hair removal and disinfection with 70% alcohol. Wounded mice were randomly divided into two groups (20 per group) and treated with normal saline and KFX three times per day. At 0–8 days, we photographed and measured the mice’s wound areas using ImageJ software. The wound healing rate was calculated as follows:

$$\text{wound reduction rate }\left(\text{\%}\right)\;=\;\left({\text{A}}_{\text{0}}-{\text{l}}_{\text{t}}\right)\text{/}{\text{A}}_{\text{0}}\text{ × }100\text{\%}$$

where A_0_ was the initial wound area (T_0_) and l_*t*_ was the wound area at the time interval (*t* ≥ 1). We used ImageJ software in accordance with the instructions offered by Schneider (Nature Methods, 2012).

### Hematoxylin and eosin staining

We fixed the traumatized cutaneous tissues in 4% paraformaldehyde for 24 h and then prepared tissue paraffin slides. The slides were dewaxed, rehydrated, and washed with different reagents before staining with hematoxylin and eosin (H&E; Beyotime Biotech). We obtained pictures under a bio-electron microscope (DP80; Olympus Corp., Tokyo, Japan). Picture magnification was ×100. We analyzed wound size, granular-tissue thickness, and hair follicle regeneration.

### Immunohistochemistry and immunofluorescence

We fixed the traumatized cutaneous samples in 4% paraformaldehyde for 24 h and then prepared tissue paraffin slides. After dewaxing and dehydration, antigen retrieval was performed at 95°C for 10 min. We blocked the slides with 5% BSA at room temperature (RT) for 1 h. Anti-vimentin primary aB was diluted to 1:1000, anti-K5 primary aB to 1:1500. After rinsing them with PBS, we incubated the slides with the secondary aB for 1 h. The slides were then counterstained with DAPI to stain the nuclei. Finally, we photographed them under a bio-electron microscope (BX53; Olympus Corporation, Tokyo, Japan).

For immunofluorescence (IF), fluorescein isothiocyanate (FITC)–labeled fluorescent primary aBs against PCNA (dilution, 1:500) and vimentin (dilution, 1:1000) were incubated overnight at 4°C. On day 2, we rewarmed and washed the sections. Sections were incubated with Alexa Fluor 488 fluorescent secondary antibodies (dilution, 1:1000) in the dark for 1 h. After counterstaining them with DAPI, we observed the slides under a fluorescence microscope and photographed them. Three fields of view were analyzed; picture magnification was ×100. ImagePro Plus v6.0 was used to analyze the experimental data and calculate average optical density (OD). We estimated the percentage of double-fluorescent–labeled cells among ECs or MSCs using different fluorescence-labeled cell counting methods and fluorescence intensity statistics in ImageJ. In addition, we anonymously shuffled image order statistics to prevent statistical preferences. We estimated the percentage of double-fluorescent–labeled cells among either epithelial or stromal cells using ImagePro Plus v6.0.

### RNA extraction and real-time polymerase chain reaction

Total traumatized cutaneous-tissue RNA was extracted using an RNA Fast Kit (Yeasen Biotechnology, Shanghai, China). We synthesized cDNA using a HiScriptII Reverse Kit (Roche). Real-time polymerase chain reaction (RT-qPCR) was performed using FastStart Universal SYBR Green PCR Mix on an IQ5 PCR System (BioRad Laboratories, Hercules, CA, United States). The reaction procedure was as follows: 40 cycles of 94°C for 1 min, 94°C for 20 s, 58°C for 30 s, and 72°C for 30 s. Primers used are listed in Table 1. We calculated mRNA expression of related target genes using the classic formula 2^−xpre^. Glyceraldehyde 3-phosphate dehydrogenase (GAPDH) was used as the internal gene.

**TABLE 1:** Reagents.

Name	Catalog no.	Manufacturer
MedGro Stem Cell Medium	PM-6105B	PUMA Bio, Shanghai, China
CCK-8	A5015	Yoshi Technology Co. Ltd., Shenzhen, China
Trypsin–EDTA	25300-054	GIBCO (Thermo Fisher Scientific, Waltham, MA, United States)
Avertin anesthetic	T4840-2	MilliporeSigma, St. Louis, MO, United States
Hematoxylin	MS4008	Beyotime Biotech, Nantong, China
RIPA	R0278	MilliporeSigma, St. Louis, MO, United States
PMSF	P7626	MilliporeSigma, St. Louis, MO, United States
Collagenase II	C6885	MilliporeSigma, St. Louis, MO, United States
Collagenase IV	C5138	MilliporeSigma, St. Louis, MO, United States
BSA	A7030	MilliporeSigma, St. Louis, MO, United States
Paraffin wax	8002-74-2	Thermo Fisher Scientific, Waltham, MA, United States
DEPC	D5758-25ML	MilliporeSigma, St. Louis, MO, United States
TriZol	15596026	Invitrogen, Carlsbad, CA, United States
Anti-fade reagent	P36934	Invitrogen, Carlsbad, CA, United States
IHC primary-aB diluents	D608501	Sangon Biotech, Shanghai, China
Western primary-aB diluents	ER0691	Sangon Biotech, Shanghai, China
DAB Color Kit	SA1023	Boster, Wuhan, China
Sodium citrate antigen repair agent	C1032	Boster, Wuhan, China
Goat serum	AR0009	Boster, Wuhan, China
Concentrated BCA protein	C1052	Beyotime Biotech, Nantong, China
Nonfat milk powder	ZY130907	MilliporeSigma, St. Louis, MO, United States
Protein marker	26616	Thermo Fisher Scientific, Waltham, MA, United States
RNA extraction kit	DP315	Tiangen Biotech Co., Ltd., Beijing, China
PVDF membrane	ISEQ00010	MilliporeSigma, Burlington, MA, United States
FastStart Universal SYBR Green	4913914001	Roche Holding AG, Basel, Switzerland
cDNA reverse transcription kit	04897030001	Roche Holding AG, Basel, Switzerland
DAPI	D1306	Thermo Fisher Scientific, Waltham, MA, United States
Anti-vimentin aB	ab92547	Abcam, Cambridge, United Kingdom
Anti–α-SMA aB	A5228	MilliporeSigma, St. Louis, MO, United States
Anti-PCNA aB	CST2586	CST, Beverly, MA, United States
Anti-K5 aB	ab24647	Abcam, Cambridge, United Kingdom
Goat anti-rabbit Alexa Fluor 888	A11008	Invitrogen, Carlsbad, CA, United States
Goat anti-rat Alexa Fluor 888	A11006	Invitrogen, Carlsbad, CA, United States
Donkey anti-goat Alexa Fluor 888	A11055	Invitrogen, Carlsbad, CA, United States
Goat anti-mouse Alexa Fluor 888	A11001	Invitrogen, Carlsbad, CA, United States

CCK-8, Cell Counting Kit-8; EDTA, ethylenediaminetetraacetic acid; RIPA, radioimmunoprecipitation assay; PMSF, phenylmethylsulfonyl fluoride; BSA, bovine serum albumin; DEPC, diethyl pyrocarbonate; IHC, immunohistochemical; aB, antibody; DAB, 3,3′-diaminobenzidine; RNA, ribonucleic acid; PVDF, polyvinylidene difluoride; cDNA, complementary deoxyribonucleic acid; DAPI, 4′,6-diamidino-2-phenylindole; α-SMA, alpha smooth-muscle actin; PCNA, proliferating cell nuclear antigen; CST; Cell Signaling Technologies.

### Transcriptomics

The samples used in the transcriptomics experiment were the suckling mouse MSCs treated with different concentrations (0, 0.05, 0.1, 0.2, and 0.25 mg/ml) of KFX for 24 h. Transcriptomics sequencing was performed by Majorbio Co., Ltd. (Shanghai, China). Briefly, RNA quality and integrity were assessed using a NanoDrop 2000 system (Thermo Fisher). We used 2 µg RNA per sample as the input control for transcriptomics sequencing. A clustering analysis was performed using a cBot Cluster Generation System and a TruSeq PE Cluster Kit v3-cBot-HS (Illumina, San Diego, CA, United States). Genes with *p* < 0.05 were considered to be differentially expressed.

### Western blotting

We collected wound tissues for total protein extraction and analyzed STAT3 and p-STAT3 levels after 3 days of cutaneous-wound healing *in vivo*. Traumatized cutaneous tissues from mice were scraped and resuspended in 0.5 ml RIPA; tissue lysates were placed on ice for 1 h. After centrifugation at 10,000*g* for 20 min, we determined total protein concentration using a Bicinchoninic Acid (BCA) Protein Determination Kit (Yeasen). We electrophoresed 20-ct samples *via* 8% sodium dodecyl sulfate–polyacrylamide gel electrophoresis (SDS-PAGE; Yeasen) and transferred them onto the PVDF membrane. Samples were blocked with blocking buffer at RT for 2 h and then probed with STAT3 (dilution, 1:500; Invitrogen), p-STAT3 (dilution, 1:1000; Invitrogen), and β-actin (dilution, 1:10000; AB clonal Technology, Woburn, MA, United States) at 4°C overnight. Secondary aBs used for detection included horseradish peroxidase (HRP)–conjugated anti-rabbit immunoglobulin G (IgG; dilution, 1:10000; ABclonal Technology). We detected target protein expression using an Enhanced Chemiluminescence (ECL) Detection Kit (Boster).

### Statistical analysis

We used the classic scientific software SPSS version 17.0 (IBM Corp., Armonk, NY, United States) for statistical-data analysis. Data were uniformly represented as mean ± standard deviation (SD). We compared different groups using Student’s *t* test and one-way analysis of variance (ANOVA). *p* < 0.05 was considered to indicate a statistically significant difference. We plotted the analyzed data using GraphPad Prism software version 8.0 (GraphPad Software, Inc., San Diego, CA, United States).

## Results

### Kangfuxin could promote cutaneous–epithelial-cell proliferation and migration *in vitro*

**FIGURE 1:**
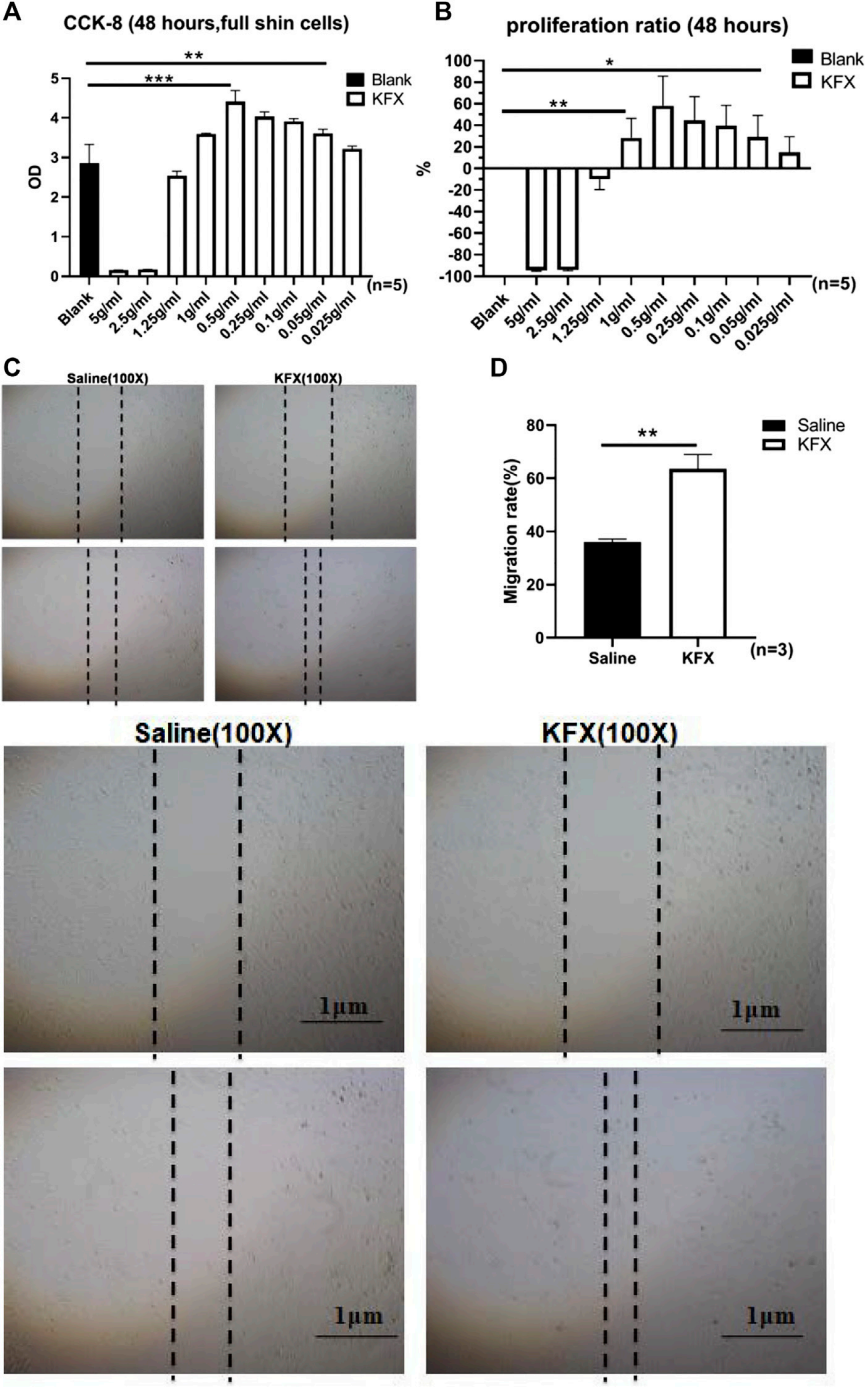
Effects of different doses of Kangfuxin on proliferation and migration of cutaneous epithelial cells at 48 h **(A,B)** OD values and proliferation ratio of cutaneous ECs after treatment with different doses of KFX for 48 h **(C,D)** Wound healing assay images and migration rate data obtained at 48 h after cell scratching. Data are shown as mean ± SD values. *p < 0.05, **p < 0.01, ***p < 0.001 vs. control group. NS, not significant.

Cell proliferation and migration are necessary to restore cutaneous integrity and accelerate the growth of ECs at the cutaneous-wound site *via* epidermal-cell migration and proliferation from the wound tissue edge (Qian et al., 2016). We used a CCK-8 assay to confirm cutaneous-EC proliferation in the presence of KFX at 48 h. Compared with the control group, the results showed that KFX could stimulate proliferation of cutaneous ECs in a dose-dependent manner. A low dose (0.5 g/ml, 5%) induced optimal growth in cutaneous ECs at 48 h (Figures 1A,B). Conversely, high doses of KFX (5 and 2.5 mg/ml) inhibited cutaneous-EC proliferation and showed a steep, dose-dependent dominant toxicity. Furthermore, KFX treatment caused evident cell proliferation after 48 h. We then used a scratch wound assay to assess cutaneous-EC migration *in vitro*. Figures 1C,D shows that, compared with the control group, KFX significantly improved cell wound closure migration in the KFX-treated group (*p* < 0.05). Taken together, the above results indicated that KFX could promote the proliferation and migration of cutaneous ECs *in vitro*.

### Kangfuxin significantly promoted expression of genes related to angiogenesis and cell adhesion

**FIGURE 2:**
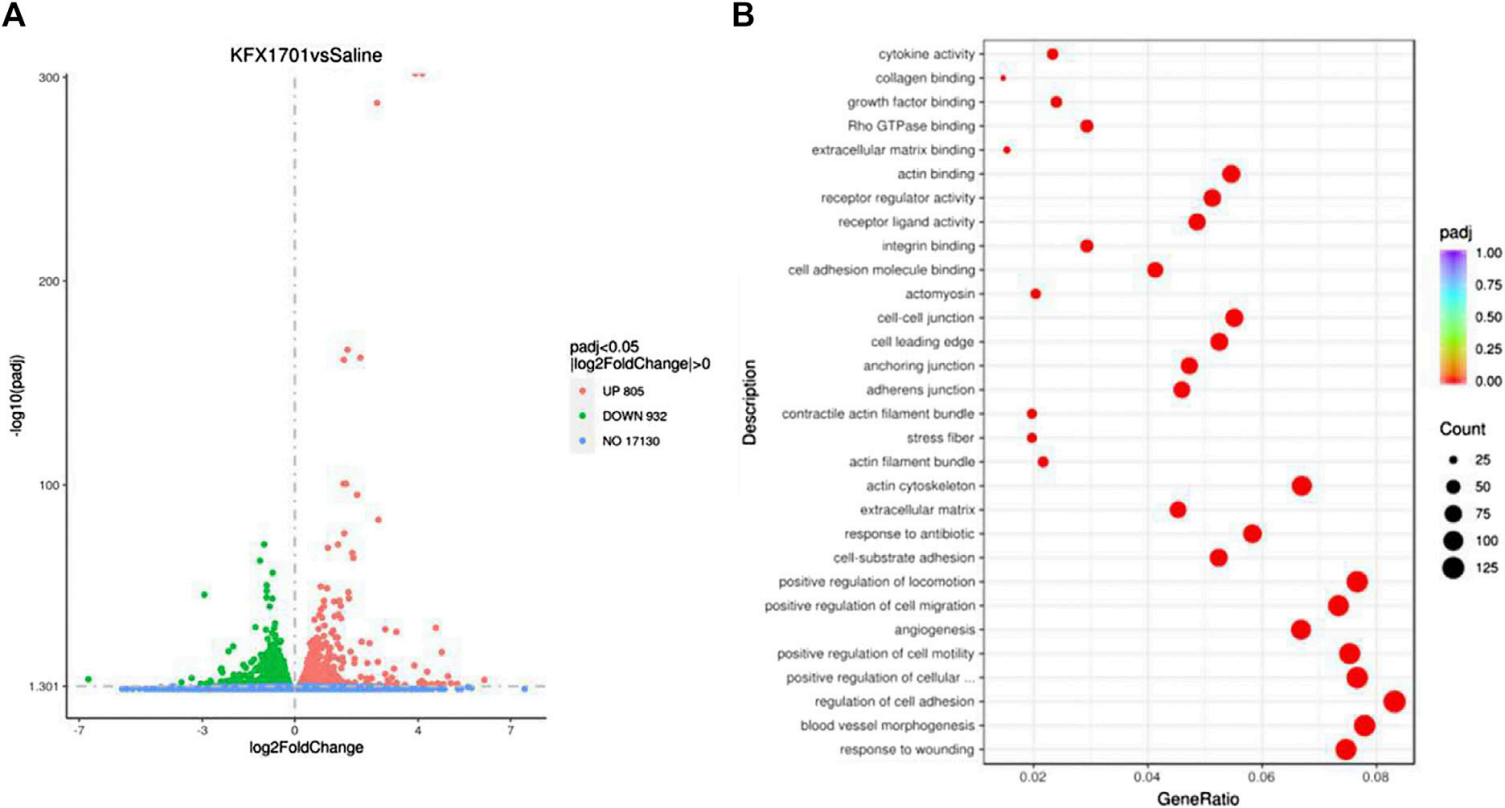
Transcriptomics sequencing analysis of Kangfuxin’s effect on cutaneous epithelial cells. **(A)** Volcano plot. **(B)** Kyoto Encyclopedia of Genes and Genomes (KEGG) enrichment scatterplot.

To further determine the effects of KFX on cutaneous ECs, we performed transcriptomics sequencing analysis on cutaneous ECs treated with KFX. The results showed that, compared with the saline group, KFX could upregulate 805 genes and downregulate 932 (Figure 2A). The top 20 most up- and downregulated genes were as follows: *Upregulated:* matrix metalloproteinase 1 (*Mmp3*), prostaglandin reductase 1 (*Ptgr1*), heme oxygenase 1 (*Hmox1*), decorin (*Dcn*), glucose-6-phosphate-dehydrogenase (*G6pd*), aryl hydrocarbon receptor repressor (*Ahrr*), 2,3,7,8-tetrachlorodibenzo-*p*-dioxin (TCDD)–inducible poly (adenosine diphosphate [ADP]–ribose) polymerase (*Tiparp*), thioredoxin reductase 1 (*Txnrd1*), solute carrier family 48 member A1 (*Slc48a1*), cystatin 6 (*Cst6*), KIT ligand (*Kitlg*), *Fosl1*, vascular endothelial growth factor delta (*Vegfd*), arginine vasopressin receptor 1A (*Avpr1a*), *Mmp13*, scavenger receptor class A member 5 (*Scara5*), glutamate–cysteine ligase catalytic subunit (*Gclc*), chemokine (C-C motif) ligand 2 (*Ccl2*), glutaredoxin (*Glrx*), and cyclin-dependent kinase inhibitor 1a (*Cdkn1a*). *Downregulated:* calponin 1 (*Cnn1*), myosin-11 (*Myh11*), actin alpha 2 (*Acta2*), secreted phosphoprotein 1 (*Spp1*), glypican 4 (*Gpc4*), *Acta1*, T-box transcription factor 18 (*Tbx18*), coiled-coil domain containing 80 (*Ccdc80*), serine protease 23 (*Prss23*), transforming growth factor-beta 3 (*Tgfb3*), actin filament–associated protein 1l2 (*Afap1l2*), gamma-actin 2 (*Actg2*), lymphocyte cytosolic protein 1 (*Lcp1*), *Slc6a6*, keratin 7 (*Krt7*), angiomotin (*Amot*), growth arrest specific 6 (*Gas6*), tropomyosin 2 (*Tpm2*), and *Myh10*. Our analysis found that upregulated expression was mainly concentrated in genes related to cell adhesion and angiogenesis, as shown in Figure 2B. Therefore, KFX significantly promoted the expression of genes related to these processes.

### Kangfuxin could promote cutaneous-wound healing *in vivo*

**FIGURE 3:**
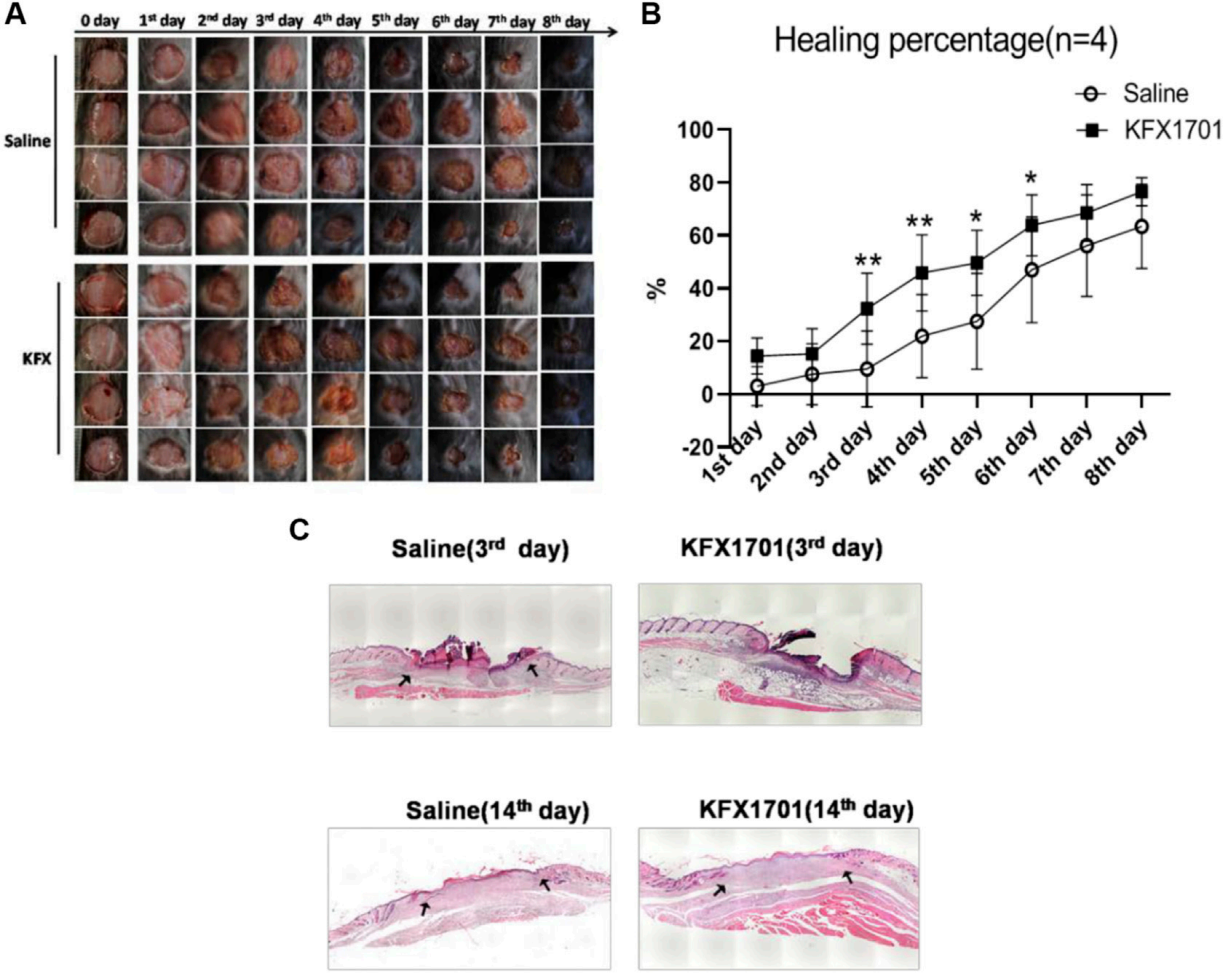
*In vivo* wound-healing effect of Kangfuxin in a cutaneous-perforation wound-healing animal model. **(A)** Photographs of wounds taken 0–8 days after cutaneous-perforation wounding in the saline and KFX groups. Scale bar = 5 mm. **(B)** Wound healing is indicated by the reduction in percentage of the initial wound area at 1–8 days after cutaneous-perforation wounding. Data are shown as mean ± SD values. *p < 0.05, **p < 0.01, ***p < 0.001 vs. the control group. NS, not significant. **(C)**, H&E staining of cutaneous-wound tissues in KFX group and saline group

We evaluated the beneficial activities of KFX in cutaneous-wound healing in a cutaneous-perforation wound healing animal model. Compared with the untreated group, the KFX formulation promoted cutaneous healing 3 days after initial wound information. On days 3–6 after cutaneous-perforation wounding, the wound area in the KFX treatment group was obviously smaller than that in the saline group (Figure 3A). Interestingly, the KFX group achieved complete wound healing within 8 days after perforation wounding, indicating that this group experienced a far better subsequent wound healing effect than the saline group (Figures 3A,B). Furthermore, H&E staining of cutaneous-wound tissues (Figure 3C) showed that epidermal tissues were remarkably thicker in the KFX group than in the saline group at days 3 and 14 after cutaneous-perforation wounding. In summary, KFX could promote cutaneous-wound healing *in vivo*.

### Kangfuxin could promote proliferation of cutaneous cells *in vivo*

**FIGURE 4:**
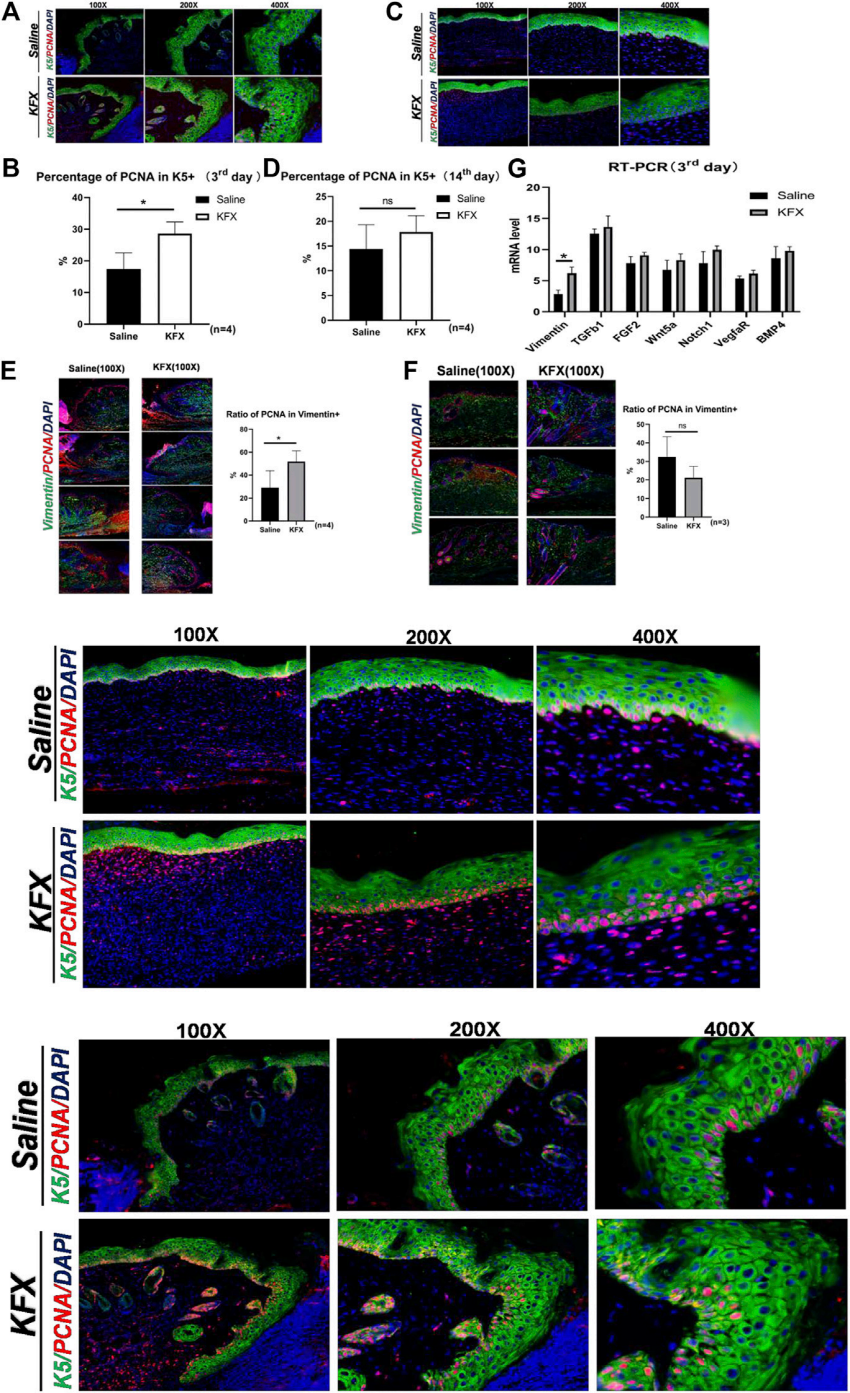
Expression of markers specific to the proliferation of cutaneous cells *in vivo* as shown by immunohistochemical and RT-qPCR analysis. **(A–D)** IHC staining of the epidermal-cell molecular marker CK5 and cell proliferation marker PCNA on days 3 and 14 after cutaneous-perforation wounding in the saline and KFX groups. **(E–F)** IF staining of the MSC-specific marker vimentin and of PCNA on days 3 and 14 after cutaneous-perforation wounding in the saline and KFX groups. **(G)** Quantitative assessment of mRNA expression of genes related to cell proliferation and differentiation in various groups at day 3 after cutaneous-perforation wounding. The cell nucleus (blue fluorescence) was stained with DAPI. Scale bars = 100×, 200×, and 400×. Data are shown as mean ± SD values. *p < 0.05, **p < 0.01, ***p < 0.001 vs. the control group. NS, not significant.

To better understand the effect of KFX treatment on wound healing, we analyzed cutaneous-wound tissues using IHC and IF staining. Cytokeratin 5 (CK5) is an epidermal-cell molecular marker (Park et al., 2019). In our study, IHC staining was used to study epidermal regeneration of healed skin. We identified epidermal cells stained with green fluorescence as CK5 epidermal cells, while PCNA was labeled with red fluorescence to show total proliferating cells (Pan et al., 2021) (Figures 4A–D). Compared with the saline group, the KFX group saw a significant increase in K5^+^ cell count, and its PCNA^+^ cell count was also greater than that of the control group on days 3 and 14 after cutaneous-perforation wounding. These results indicated that KFX could promote epidermal-cell proliferation, differentiation, and formation in wounds.

Furthermore, cutaneous-wound healing is related to the proliferation and differentiation of mesenchymal cells, of which vimentin is a biomarker (Paulin et al., 2022). Therefore, we further analyzed the effect of KFX on the proliferation of cutaneous mesenchymal cells in wounds, using IF staining to indicate vimentin^+^ cells. Cells labeled green were considered vimentin^+^, and those co-labeled red and green indicated total proliferating-cell count among vimentin^+^ cells. As seen in Figures 4E,F, our results showed that compared with the saline group, the counts of vimentin– and PCNA–double-positive cells in the KFX group were significantly increased. Vimentin anchors and supports organelles in the cytoplasm of mesenchymal cells; its expression is upregulated during epithelial–mesenchymal transformation (EMT), which occurs during wound healing. The increase in vimentin^+^–cell count in the KFX group might be a biomarker of mesenchymal-origin cells or EMT-producing cells. In addition, we quantified mRNA levels of genes related to cell proliferation and differentiation at day 3 after cutaneous-perforation wounding using RT-qPCR. The mRNA expression of related target genesis is shown in Figure 4G. The results indicated that, relative to GAPDH, expression of vimentin was higher on day 3 after cutaneous-perforation wounding followed by KFX treatment. This indicated that KFX could promote MSC proliferation and differentiation in the wound and contribute to wound healing, and that it could also promote proliferation of cutaneous cells *in vivo*.

### Kangfuxin could enhance the signal transducer and activator of transcription 3 signaling pathway *in vivo*

**FIGURE 5:**
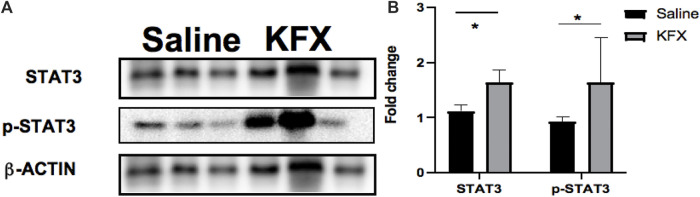
Effects of Kangfuxin on the cell proliferation and migration–related signaling pathway *in vivo*. **(A,B)** Protein levels of STAT3 signaling pathway–related proteins. β-actin protein was used as an internal control. Data are shown as mean ± SD values. *p < 0.05, **p < 0.01, ***p < 0.001 vs. the control group. NS, not significant.

To better understand the molecular mechanism of KFX-mediated regulation in the cutaneous-wound healing model, we assessed the signaling pathway associated with the proliferation and migration. Expression levels of the STAT3 signaling pathway–related proteins STAT3 and p-STAT3 were markedly upregulated in our mouse model after KFX treatment (Figures 5A,B), indicating that KFX could promote cutaneous-wound healing by enhancing the STAT3 signaling pathway *in vivo.*

## Discussion

The excellent effect of KFX on cutaneous-wound healing was fully demonstrated in this study, suggesting that KFX has great clinical potential as a substitute for skin grafting and other tissue GFs in the treatment of cutaneous wounds. To the best of our knowledge at present, KFX has not been shown to have toxic or other side effects. It is widely used to treat dermatological diseases and has shown good effects in the treatment of digestive-system diseases. Therefore, KFX is a relatively safe drug for cutaneous-wound healing. However, we did find that dominant toxic effects of KFX existed at doses five times that of the putatively therapeutic dose we used *in vitro*, as assessed by CCK-8 assay. The reason for this might be that cells in a single culture are more sensitive to drugs.

However, the molecular mechanism by which KFX regulates cutaneous-tissue reconstruction–related cells and promotes cutaneous-wound healing is still unclear. In this study, we prepared KFX to determine this molecular mechanism. We designed a cutaneous-wound healing model to demonstrate that this extract of *P. americana* promoted cutaneous-EC proliferation and migration and cutaneous-wound healing in a mouse model. The STAT3 signaling pathway was confirmed as a critical molecular mechanism in cutaneous-wound healing promoted by KFX. In this study, expression levels of STAT3 and p-STAT3 were markedly upregulated in our animal model after KFX treatment, indicating that KFX could promote cutaneous-wound healing by enhancing the STAT3 signaling pathway *in vivo.* Ours study links the molecular mechanism of KFX to the STAT3 signaling pathway. In order to verify the specificity of this in cutaneous-wound healing, it is necessary to detect the therapeutic effect of KFX by inhibiting the pathway; this research is still in progress. Furthermore, this study is the first to report the effect of KFX on the proliferation of ECs and skin interstitial cells in the treatment of cutaneous wounds.

There are many reasons for poor cutaneous-wound healing. It can be related to infectious factors and to extant underlying diseases such as diabetes, cirrhosis, nephropathy, and malignant tumors (Wang et al., 2020). In addition, anemia, malnutrition, old age, physiological-function decline, and obesity can facilitate poor cutaneous-wound healing (Wells and Watt, 2018). According to the theories of TCM, “poison, rot, blood stasis, and deficiency” are the basic pathologies underlying poor cutaneous-wound healing (Ramadan et al., 2019). We believe that such “deficiency” and “stasis” are mutually causal, in that qi deficiency aggravates blood stasis, blood stasis hinders qi and blood movement, and qi is left unhealthy for a long time; thus, the wound loses nourishment, and the ulcer is prolonged and does not heal (Liu et al., 2019). TCM, which has a long history of understanding and treating poor cutaneous-wound healing, has put forward the principles of “nourishing deficiency and removing blood stasis” and “removing the muscle growth and muscle leveling and skin lengthening” (Jensen et al., 2010). However, cutaneous-wound healing involves tightly regulated aspects of bodily metabolic activity, including tissue coagulation and hemostasis, inflammatory regulation, proliferation of various cell types, chemotaxis, and cell migration (Liu et al., 2015). As we have come to understand the biological mechanism of cutaneous-wound healing, the corresponding molecular mechanism has been elucidated at the cellular and gene levels. Promotion of wound healing and tissue regeneration by drugs has become a worldwide research hotspot.

KFX has been used and studied in clinical practice for decades. It is mainly used for burns, scald wounds, multiple organic ulcers, and mucosal injuries (Rao et al., 2016). The main ingredient of KFX is dried extract of the insect *P. americana*, the largest insect in the cockroach family. It is recorded in *Shennong Ben Cao Jing* that “the main blood stasis disease is firm, cold and heat, broken accumulation, with laryngeal obstruction, and no offspring in the internal cold” (Usman et al., 2021). KFX mainly contains polypeptides, amino acids, nucleosides, and alkaloids, which improve blood circulation, nourish yin, and build muscle (Yin et al., 2020). Modern pharmacological studies suggest that KFX can significantly increase counts of neutrophils, macrophages, and lymphocytes on the wound surface; improve their spontaneous and chemotactic movement; promote shedding of necrotic tissue; and create conditions for tissue repair (Fernanda de Mello Costa et al., 2020). At the same time, it can upregulate glutamine levels; stimulate protein synthesis; and provide nutritional support for mucosal cells, fibroblasts, and other rapidly proliferating cells (Cheng and Eriksson, 2017). Previous studies have shown that KFX can effectively promote angiogenesis and granular-tissue growth, improve wound microcirculation, accelerate tissue repair and regeneration, eliminate edema, and enhance the immune function, which can help repair and heal ulcers as soon as possible, protect the ulcer surface from infection, and avoid necrosis (Chigurupati et al., 2013; Evtushenko et al., 2021).

Many clinical applications have proven that KFX can promote wound healing. Relevant studies on the mechanism of *P. americana* extract have been published in recent years. Tan et al. showed that the extract can reduce expression levels of inflammatory factors *in vivo*, including Cluster of Differentiation 3 (CD3), CD4, CD8, and interleukin-2 (IL-2), thus regulating the immune function and accelerating oral-mucosa healing in rats (Govindaraju et al., 2019). Yang et al. suggested that *P. americana* extract might regulate the release of transforming growth factor β1 (TGF-β1), promote wound healing, and avoid scar formation (Liu et al., 2015; Hu et al., 2019). The experimental results of Zeng et al. showed that the extract could improve the expression level of VEGF in the wound and promote wound healing (Wells and Watt, 2018; Rong et al., 2019; Wang et al., 2020). However, the complete molecular mechanism underlying KFX in wound healing and regeneration remains unclear, especially in terms of KFX’s effect on MSCs. In our study, we explored such effect in wound healing, identified potential biological effects of KFX on MSCs in this process, and investigated its effect on cutaneous-MSC proliferation and migration *in vitro*. The results showed that KFX could promote cutaneous-MSC proliferation and migration and had obvious chemotactic effects on the latter process. We also explored the effects of KFX on cutaneous-wound healing *in vivo* and on K5-labeled epidermal cells, vimentin-labeled mesenchymal cells, and PCNA-labeled proliferative cells in cutaneous-wound healing. The results showed that the extract could increase proliferation of K5-labeled epidermal cells and vimentin^+^ cells, indicating that it could accelerate cutaneous-wound healing by improving the proliferation of interstitial cells and cutaneous MSCs. This in turn suggested that KFX could promote cutaneous-wound healing in the inflammatory-reaction stage and the cell proliferation and reconstruction stage of these wounds.

However, obvious deficiencies remain in our research. First, the molecular mechanism of KFX that we discovered needs further discussion; its molecular target is not very clear. Second, in order to ensure the reliability of experimental conclusions, we must further confirm this mechanism *via* gene knockout or antibody-blocking experiments.

## Conclusion

KFX, an ethanol extract of *P. americana*, is an excellent clinical option in cutaneous-wound treatment. The molecular mechanism underlying its promotion of cutaneous-wound healing might involve proliferation of various cutaneous cells, chemotaxis, migration of ECs, promotion of angiogenesis, and stimulation of MSC proliferation. Herein, we determined the effect of KFX on the proliferation of ECs and skin interstitial cells in the treatment of cutaneous wounds for the first time. Our study can provide a theoretical and practical basis for the clinical application and pharmaceutical-factory production of KFX, as well as lay a theoretical foundation for future wound-healing therapies and contribute to current ones.

## Data Availability

The original contributions presented in the study are included in the article/supplementary material, further inquiries can be directed to the corresponding author. The RNA-seq data presented in the study are deposited in the NCBI repository, accession number PRJNA866707.
